# Identifying key psychological characteristics among Chinese individuals with eating disorders: an exploratory graph and network analysis

**DOI:** 10.1186/s40337-025-01348-1

**Published:** 2025-07-22

**Authors:** Liyun Zheng, Chao Chen, Darong Zhang, Xueni Li, Shuxia Geng, Qingmei Kong, Tianmei Si

**Affiliations:** 1 NHC Key Laboratory of Mental Health (Peking University), Peking University Sixth Hospital, Peking University Institute of Mental Health, National Clinical Research Center for Mental Disorders (Peking University Sixth Hospital), Beijing, 100191 China; 2 Department of Psychiatry, Jiangxi Medical College, Jiangxi Mental Hospital & Affiliated Mental Hospital, Nanchang University, Nanchang, 330029 Jiangxi China; 3 Nanchang City Key Laboratory of Biological Psychiatry, Jiangxi Provincial Clinical Research Center on Mental Disorders, Jiangxi Mental Hospital, Nanchang, 330029 Jiangxi China

**Keywords:** Eating disorders, Psychological characteristics, Chinese population, Network analysis, Exploratory graph analysis

## Abstract

**Background:**

Interventions targeting core characteristics of eating disorders (EDs) can effectively alleviate symptoms. However, it remains unclear whether these characteristics exhibit cultural specificity within the Chinese population. This study combines exploratory graph analysis (EGA) and network analysis to identify key psychological characteristics in Chinese patients with EDs.

**Methods:**

The psychological characteristics of 1,001 patients with EDs were assessed using the Eating Disorder Inventory-1 (EDI-1). Nineteen representative items were selected and categorized into different dimensions through EGA. Network analysis was then performed to identify key psychological characteristics by determining central and bridge nodes.

**Results:**

In addition to the “ED-specific” and “Non-specific” categories, an unexpected category, “Perfectionism,” was identified. Across these three categories, four key psychological characteristics were highlighted: “terrified of gaining weight,” “guilty after overeating,” “worry that feelings will get out of control,” and “must do things perfectly.”

**Conclusion:**

Beyond drive for thinness, perfectionism and emotional regulation difficulties may represent key psychological characteristics among Chinese individuals with EDs. These findings could help inform the development of culturally tailored treatment strategies for EDs in China.

## Abstract (plain language summary)

This study looked at the main psychological traits of eating disorders (EDs) in people from China to understand how treatments could better fit their needs. The researchers found that people with EDs often struggle with a strong fear of gaining weight, feeling guilty after eating too much, trouble managing emotions, and putting too much pressure on themselves to be perfect.

These traits were grouped into three categories: those directly related to eating disorders, general mental health issues, and perfectionism. Perfectionism and difficulties controlling emotions were found to be just as important as concerns about weight.

This means that treating eating disorders isn’t just about food and weight—it’s also about addressing perfectionism and emotional struggles. These findings could help create more supportive and effective treatments for people in China, helping them feel more in control and less overwhelmed in their daily lives.

## Background

Eating disorders (EDs) are characterized by abnormal eating or weight control behaviors, seriously impacting both the physical and mental health of patients, and may lead to death [1]. The prevalence and burden of EDs are increasing worldwide [2, 3]. While the burden of EDs is greater in Western countries, the rate of increase is more pronounced in East Asian countries, with an estimated annual percentage change (EAPC) for prevalence at 2.23 and for disability-adjusted life years (DALYs) at 2.22 [4]. From 1990 to 2019, China experienced significant increases in the age-standardized incidence, prevalence, and DALY rates of eating disorders, with EAPCs of 1.3, 1.6, and 1.6 for anorexia nervosa, and 1.4, 2.0, and 2.0 for bulimia nervosa, respectively [5]. These upward trends may reflect not only a true increase in disease burden, but also improvements in public awareness and diagnostic capacity related to EDs, thereby indicating a rising demand for medical intervention.

Effective therapies for EDs often involve interventions targeting their key characteristics. According to Fairburn’s transdiagnostic cognitive behavioural theory, the overvaluation of body shape, weight, and their control is recognized as a key characteristic of EDs [1, 6, 7]. Recent network analysis studies have corroborated this and further identified ineffectiveness, defined as a sense of emptiness, worthlessness, insecurity, loss of control, and negative self-evaluation, and interoceptive deficits as potential key characteristics [8–12]. However, most network analysis studies focus primarily on Western populations, and key characteristics have been shown to vary across study populations [9, 13]. It remains unclear whether key characteristics differ among Chinese ED populations within distinct cultural contexts, a question critical for developing culturally tailored treatment strategies for Chinese ED groups.

Previous network analysis studies on EDs have primarily identified central nodes as core symptoms. However, focusing solely on central nodes may overlook some key characteristics. According to network theory, the interactions among symptoms collectively form the ED itself, with symptoms that have extensive connections to others being considered most critical [14, 15]. In symptom networks, bridge nodes linking different communities are just as critical as central nodes with widespread connections [14]. To our knowledge, most network analysis studies typically assign ED-related psychological characteristics to a single community without considering their potential affiliations with multiple communities (e.g., ED-specific or non-specific) [9, 16, 17]. This approach restricts the identification of potential bridge nodes.

Exploratory graph analysis (EGA) is a method that uses undirected network models to estimate the number of dimensions (i.e., communities) in multivariate data [18]. EGA assumes that the correlation between items within a community are stronger than those between items in different communities, thus potentially dividing a symptom network into distinct communities [19]. Its advantage lies in its data-driven approach, which automatically detects both the number and content of dimensions, making it relatively objective [20]. In other words, EGA provides a means of identifying potential bridge nodes within ED networks.

This study aims to identify key psychological characteristics in the Chinese population with EDs through a data-driven approach. The Eating Disorder Inventory-1 (EDI-1) will serve as an assessment tool. Key items will be selected, followed by EGA for community detection and network analysis to identify central and bridge nodes. This study is expected to identify culturally specific key psychological characteristics of EDs, which may inform more targeted treatment strategies.

## Methods

### Participants and procedure

Participants were patients aged 9–35 years with eating disorders (EDs) who attended a specialized ED treatment center comprising both outpatient clinics and inpatient wards. They were enrolled in the Eating Disorders Registration Project (ED-RP), a structured data collection initiative designed to systematically gather information from routine clinical assessments to support clinical care and treatment planning. Data were obtained through continuous enrollment, with each patient contributing data from a single assessment conducted prior to formal treatment initiation. Assessments included self-report questionnaires addressing psychological and behavioral features of EDs, as well as physical health parameters. Inclusion criteria for the present study were: (1) a diagnosis of an ED according to DSM-IV criteria [21], (2) the ability to comprehend the questionnaire, and (3) Written informed consent was obtained form the patients or their legal guardians. Exclusion criteria included: (1) severe psychiatric disorders such as intellectual disability, organic mental disorders, schizophrenia, or bipolar disorder, (2) self-harm or suicidal behavior, or reported suicidal ideation, and major depressive disorder, and (3) Patients with severe physical conditions (e.g., severe malnutrition) or significant abnormal findings during routine outpatient or inpatient laboratory tests (e.g., severe anemia, markedly elevated liver enzymes), deemed unsuitable for participation by physicians, were excluded.

From August 2008 to October 2015, 1,056 patients, including 992 females and 64 males, participated after screening based on the inclusion and exclusion criteria. Diagnoses were confirmed by two specialists through psychiatric interview. Questionnaires were given to patients during outpatient visits or before inpatient admission. Height and weight were measured on-site and noted on the questionnaire cover page. Subsequently, patients completed the paper-based questionnaire in the outpatient or inpatient reception area under the supervision of trained research staff. Clarification was provided when necessary to ensure full comprehension of item content; however, no guidance or prompting was given. Participation was entirely voluntary, and all assessments were conducted as part of routine clinical care when clinically appropriate. Patients could withdraw from the study at any stage without impact on their clinical care. After excluding 55 patients who either did not complete the questionnaire or declined research use of their data, a final sample of 1,001 patients was included in the analysis.

### Measures

To assess the psychological characteristics of patients with EDs, we selected the Eating Disorder Inventory-1 (EDI-1) scale [22]. EDI-1 is recognized as an essential tool for the multidimensional evaluation of ED-related psychopathology [23]. The Chinese version of the EDI-1 demonstrates good reliability and validity, except for the Maturity Fears subscale (Cronbach’s α = 0.50) [24]. EDI-1 comprises 64 items divided into eight subscales. Subscales such as Drive for Thinness, Bulimia, and Body Dissatisfaction primarily assess specific psychological characteristics of EDs, including attitudes towards eating-related behaviors, weight, and body shape [25]. Conversely, the Ineffectiveness, Perfectionism, Interpersonal Distrust, Interoceptive Awareness, and Maturity Fears subscales assess broader psychological characteristics associated with EDs but not specific to EDs [25]. Participants rate each item on a six-point scale from 1 (never) to 6 (always). As item-level analysis was prioritized following completion of the EDI-1 to gain more detailed insight into specific psychological characteristics, subscale recoding was not conducted [13].

### Statistical analysis

#### Missing data

Given the minimal proportion of missing data (0.1–0.5%) and the absence of systematic patterns across key variables, a Missing At Random (MAR) mechanism was assumed. Multiple imputation was then performed using the *mice* package in R to generate five imputed datasets. The final dataset was derived by calculating the rounded average across these datasets.

#### Node selection

In network analysis, it is necessary to remove items with overlapping meanings to avoid the expansion of node centrality [26]. For this purpose, we referred to the 27 items selected by Schlegl et al. through a focus group approach from the 11 subscales of the EDI-2 [13]. These items were selected based on their distinctiveness and ability to represent key constructs within each subscale. Given that the first eight subscales of the EDI-2 were fully consistent with the EDI-1, we retained the corresponding 19 items from these subscales for network analysis. To ensure non-redundancy at the item level, we applied the goldbricker function, which confirmed the absence of topological overlap among the selected nodes [27].

#### Exploratory graph analysis

The EGA analysis was conducted using the EGA*net* package in R (4.3.1) [28]. Items are mapped into a network consisting of nodes and edges, where nodes represent the items and edges indicate the strength of interaction between the items. By applying penalized inverse covariance matrices, EGA effectively removes spurious associations and preserves genuine relationships, thereby classifying items based on their affinity. EGA estimates the correlation matrix of items using graphical LASSO (Least Absolute Shrinkage and Selection Operator) to avoid overfitting. The number of dimensions was verified using the walktrap method, which visualizes the dimensional structure by measuring node similarity through random walks. Additionally, EGA provides a bootstrap function to evaluate the stability of the number of dimensions and the item mappings [29]. In this study, 1000 bootstrap samples were drawn, and the stability threshold for item placement on dimensions was set at 0.70 [30].

#### Network Estimation

Network construction and visualization were performed using the *qgraph* package in R [31]. The constructed network was undirected and weighted, where nodes represented various items from the EDI-1. Edges, the lines connecting nodes, represent partial correlations between these dimensions. The magnitude of these correlations was indicated by the edge thickness, quantified as edge weight. The LASSO technique was applied in conjunction with the extended Bayesian information criterion (EBIC), set at a 0.5 threshold [32]. This approach effectively eliminated edges representing weaker correlations, thus refining the network’s structure.

#### Centrality indices

To comprehensively evaluate centrality within the symptom network, both strength and expected influence (EI) centrality indices were employed. The utilization of Strength, defined as the sum of the absolute values of all edge weights, provides a measure of a node’s overall connectedness in the network [33]. EI, meanwhile, extends this concept by accounting for both positive and negative edge weights, offering a more nuanced view of a node’s influence [33]. To assess bridge centrality, pivotal in understanding the interplay between ED-specific and non-specific psychological characteristics, both bridge strength and bridge EI metrics were applied. Bridge strength centrality, defined as the sum of the absolute values of edge weights connecting a node with nodes outside its cluster, is used to assess inter-cluster connections [34]. Bridge EI is like bridge strength but additionally considers both positive and negative inter-cluster connections [34]. Furthermore, to ascertain the significance of node and bridge node centrality, centrality difference tests were conducted [35]. These tests were instrumental in determining whether nodes with higher centrality differed significantly from those with lower values, thereby clarifying the significance of certain nodes or bridges in the network.

#### Network stability

To ensure the robustness and reliability of the network model, five key aspects of network stability were systematically evaluated using the R package *bootnet* [35]. These aspects included the accuracy of edge weights, stability of strength centrality, EI, bridge strength, and bridge EI. The accuracy of edge weights was assessed using 95% confidence intervals derived from each edge’s weight through non-parametric bootstrapping (with 1000 bootstrapped samples), where narrower intervals indicated higher accuracy. Furthermore, node EI stability was tested through case-dropping bootstrapping, involving 1000 bootstrapped samples. The stability of strength centrality, EI, bridge strength, and bridge EI was quantified using the correlation stability (CS) coefficient. A CS coefficient above 0.70 was classified as indicating excellent stability [35].

## Result

### Demographic data

The mean age of 1001 participants was 20.54 years with a standard deviation (SD) of 5.10 years. Regarding body mass index, the mean was 17.42 kg/m² with a SD of 3.63. The sample included 689 adult patients (aged ≥ 18 years), whose mean percentage of ideal body weight (%IBW) was 84% (SD = 17%), with ideal body weight (IBW) calculated using the Chinese modified Broca formula (IBW = height − 105) [36]. Additionally, 292 adolescent patients (aged 13–17 years) had a mean %IBW of 84% (SD = 18%), and 20 child patients (aged 9–12 years) had a mean %IBW of 88% (SD = 20%). For both adolescents and children, IBW was estimated based on age-specific median weights derived from Chinese national growth references [37].

The gender distribution of the participants was predominantly female, with 947 females (94.6%) and 54 males (5.4%). In terms of profession, most of the participants were students, accounting for 694 individuals (69.3%), while the remaining 307 (30.7%) were engaged in other professions. When considering educational background, 438 participants (43.8%) had a high school degree or less, and 533 participants (53.2%) had a university degree or higher. Marital status was also recorded, with a large majority of the participants being unmarried (889 individuals, 88.8%), while 112 participants (11.2%) were married or cohabiting. Regarding their diagnoses, 455 individuals (45.5%) were diagnosed with Anorexia Nervosa, 251 (25.1%) with Bulimia Nervosa, and 295 (29.5%) with Eating Disorder Not Otherwise Specified.

### Network dimensions

**Fig. 1:**
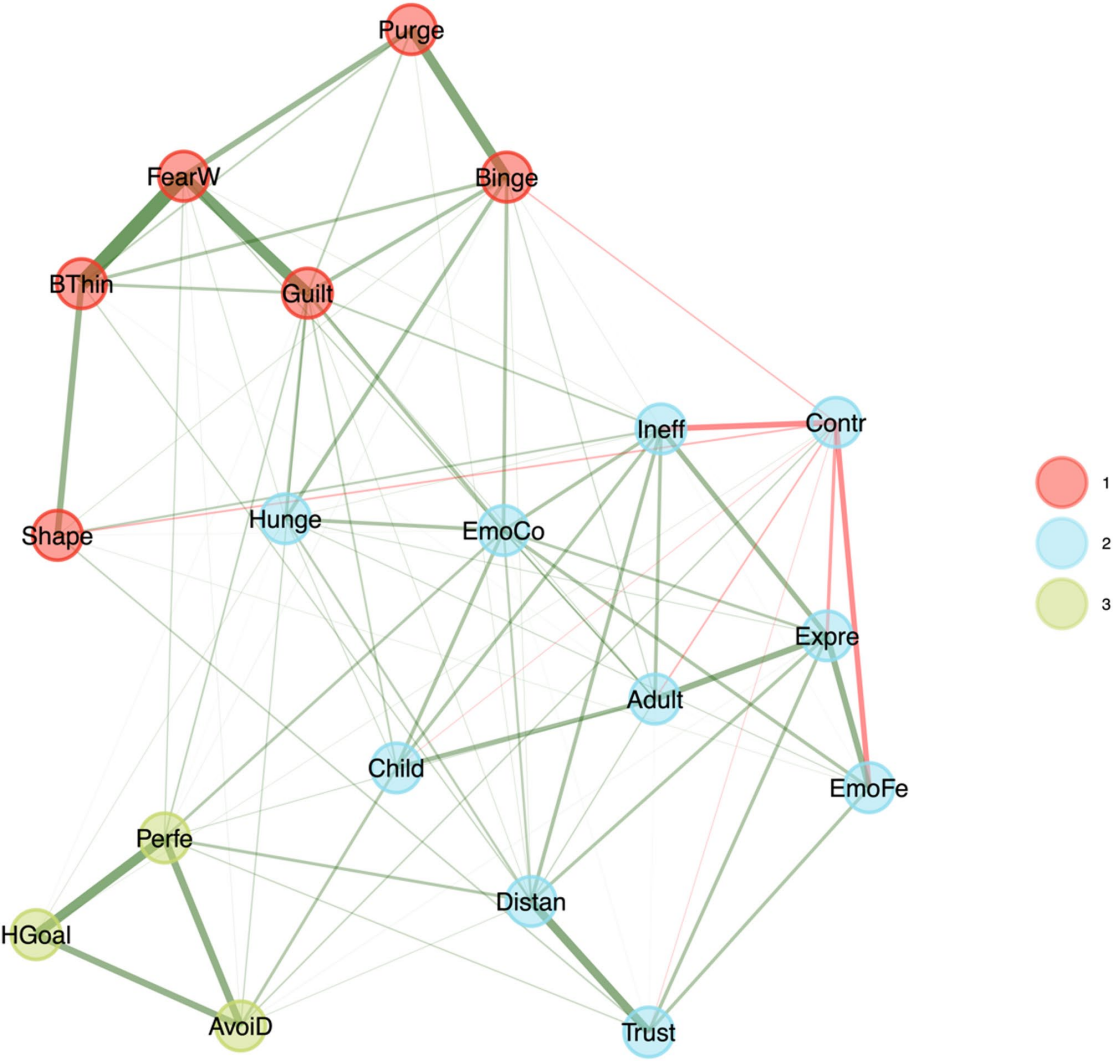
Graphical Network of eating disorders. The thickness of the edge is the degree of correlation, with positive correlations depicted as green while negative correlations depicted as red. Dimensions: 1 = “ED-specific”, 2 = “Non-specific”, 3 = “Perfectionism”

In the EGA conducted for all ED patients, the previously selected 19 items were input. The stability for each item was no less than 0.7, and no items were excluded. The median number of dimensions determined by 1000 bootstrap tests was 3 (SE = 0.20, 95% CI [2.60, 3.40]). In this structure comprising 3 dimensions and 19 items, all items were clearly assigned to one dimension, with item stability values ranging from 0.70 to 1.00. The network loading for items within each dimension ranged from 0.15 to 0.65. Dimension 1 included items 1–6, Dimension 2 included items 7–16, and Dimension 3 included items 17–19 (see Fig. 1**and** Table 1). The dimensions were labeled based on the content evaluation of the items assigned to different structures. Dimension 1 encompassed ED-specific psychological characteristics and was labeled “ED-specific.” Dimension 2 primarily included broadly present, non-specific psychological characteristics and was named “Non-specific.” Dimension 3 was labeled “Perfectionism,” as all items in this dimension were derived from the original subscale of the same name.

**Table 1:** Overview of the 19 included items of the eating disorder Inventory-1 and their stability and loadings on dimensions

No.	Item abbreviation	Short item text	Item stability	Network loading on dimensions		
				1 ED-specific	2 Non-specific	3 Perfectionism
**1**	Guilt	Guilty after overeating	1.00	**0.42**	0.22	0.04
**2**	FearW	Terrified of gaining weight	1.00	**0.65**	0.06	0.04
**3**	BThin	Desire to be thinner	1.00	**0.62**	0.03	<0.01
**4**	Binge	Could not stop eating binges	1.00	**0.37**	0.20	<0.01
**5**	Purge	Thought of trying to vomit	1.00	**0.40**	0.01	<0.01
**6**	Shape	Feel satisfied with the shape	0.81	**0.15**	0.12	<0.01
**7**	Ineff	Feel ineffective as a person	0.99	<0.01	**0.49**	<0.01
**8**	Contr	Feel generally in control	0.99	-0.06	**-0.35**	0.05
**9**	Trust	Trust others	0.99	0.03	**0.31**	0.03
**10**	Expre	Have trouble expressing emotions	0.99	<0.01	**0.60**	0.01
**11**	Distan	Keep people at a certain distance	0.99	0.07	**0.41**	0.08
**12**	EmoFe	Clearly identify what emotion feeling	0.99	0.01	**0.38**	<0.01
**13**	Hunge	Get confused as to whether or not hungry	0.70	0.14	**0.19**	0.02
**14**	EmoCo	Worry that feelings will get out of control	0.97	0.14	**0.42**	0.06
**15**	Child	Return to the security of childhood	0.97	0.04	**0.26**	0.06
**16**	Adult	Demands of adulthood are too great	0.99	0.07	**0.38**	0.02
**17**	AvoiD	Avoid disappointing parents and teachers	1.00	0.03	0.04	**0.43**
**18**	Perfe	Must do things perfectly	1.00	0.07	0.15	**0.52**
**19**	HGoal	Have extremely high goals	1.00	0.01	<0.01	**0.50**

### Network structure

**Fig. 2:**
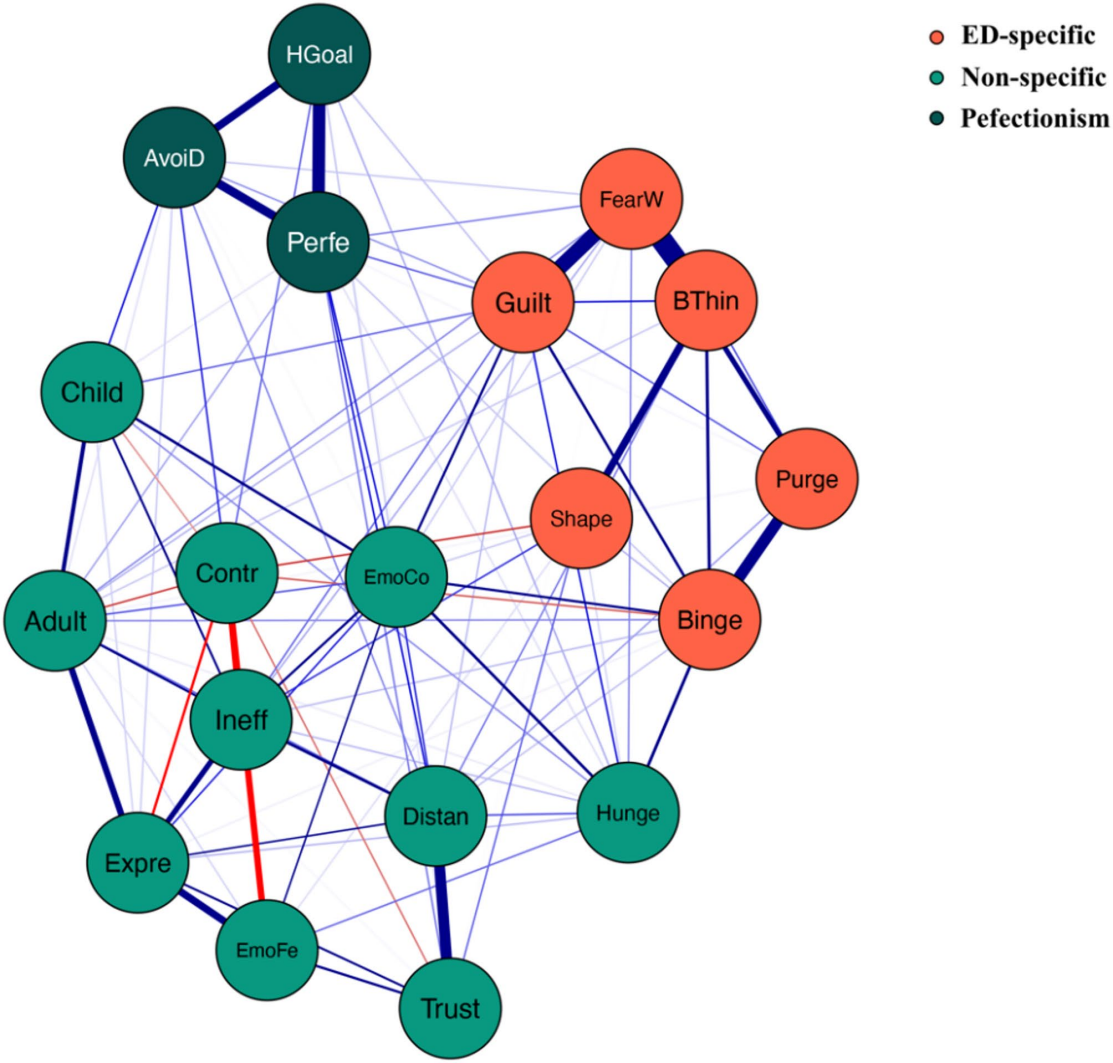
Network model includes three dimensions.,The blue edge represents positive partial correlation, the red edge represents negative partial correlation. A thicker and more saturated edge represents stronger relationship. Network labels: Guilt = guilty after overeating; FearW = terrified of gaining weight; BThin = desire to be thinner; Binge = could not stop eating binges; Purge = trying to vomit; Shape = feel satisfied with the shape; Ineff = feel ineffective as a person; Contr = feel generally in control; AvoiD = avoid disappointing parents and teachers; Perfe = must do things perfectly; HGoal = have extremely high goals; Trust = trust others; Expre = have trouble expressing emotions; Distan = keep people at a certain distance; EmoFe = clearly identify what emotion feeling; Hunge = get confused as to whether or not hungry; EmoCo = worry that feelings will get out of control; Child = return to the security of childhood; Adult = demands of adulthood are too great

**Fig. 3:**
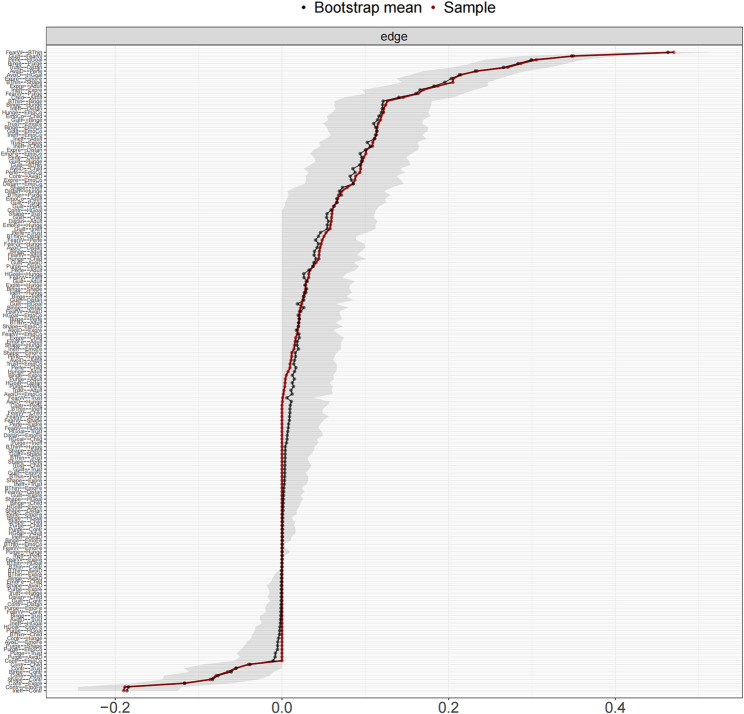
Accuracy of edge weights in the network. The red solid line depicts the sample edge weights, and the gray shaded bar depicts the bootstrapped confidence interval

**Fig. 4:**
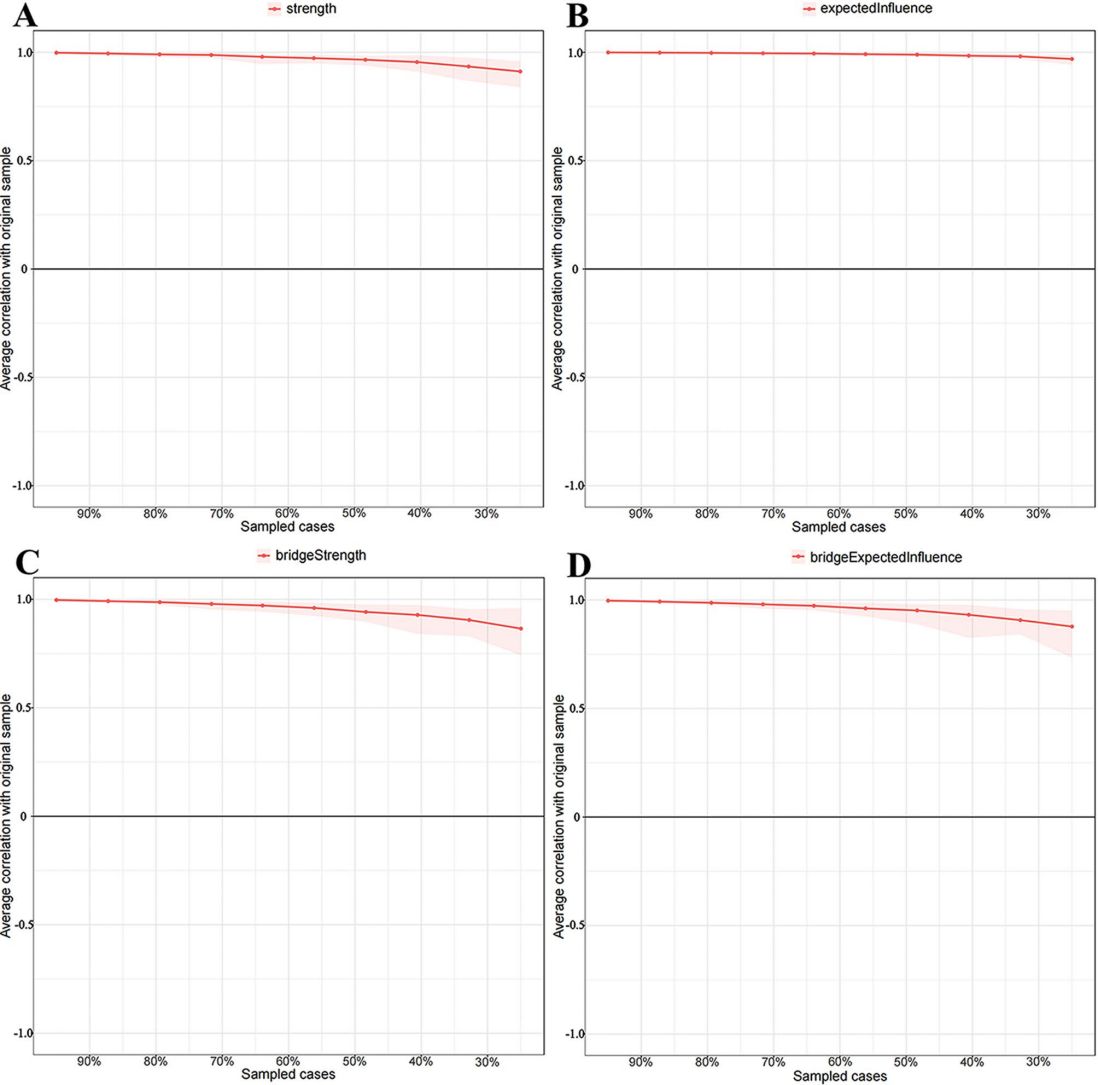
Stability of (**A**) strength, (**B**) expected influence, (**C**) bridge strength, and (**D**) bridge expected influence in the network. The red solid lines represent the average correlation between (**A**) strength, (**B**) EI, (**C**) bridge strength, or (**D**) bridge EI in the full sample and subsample with the red area depicting the 2.5th quantile to the 97.5th quantile

The network structure demonstrated stability. Figure 2 depicts the network model that includes three dimensions. A narrow 95% confidence interval for the edge weights in the network indicated high accuracy (see Fig. 3). Additionally, stability tests for strength, EI, bridge strength, and bridge EI yielded CS coefficients of 0.75 for each metric (see Fig. 4).

### Central nodes

**Fig. 5:**
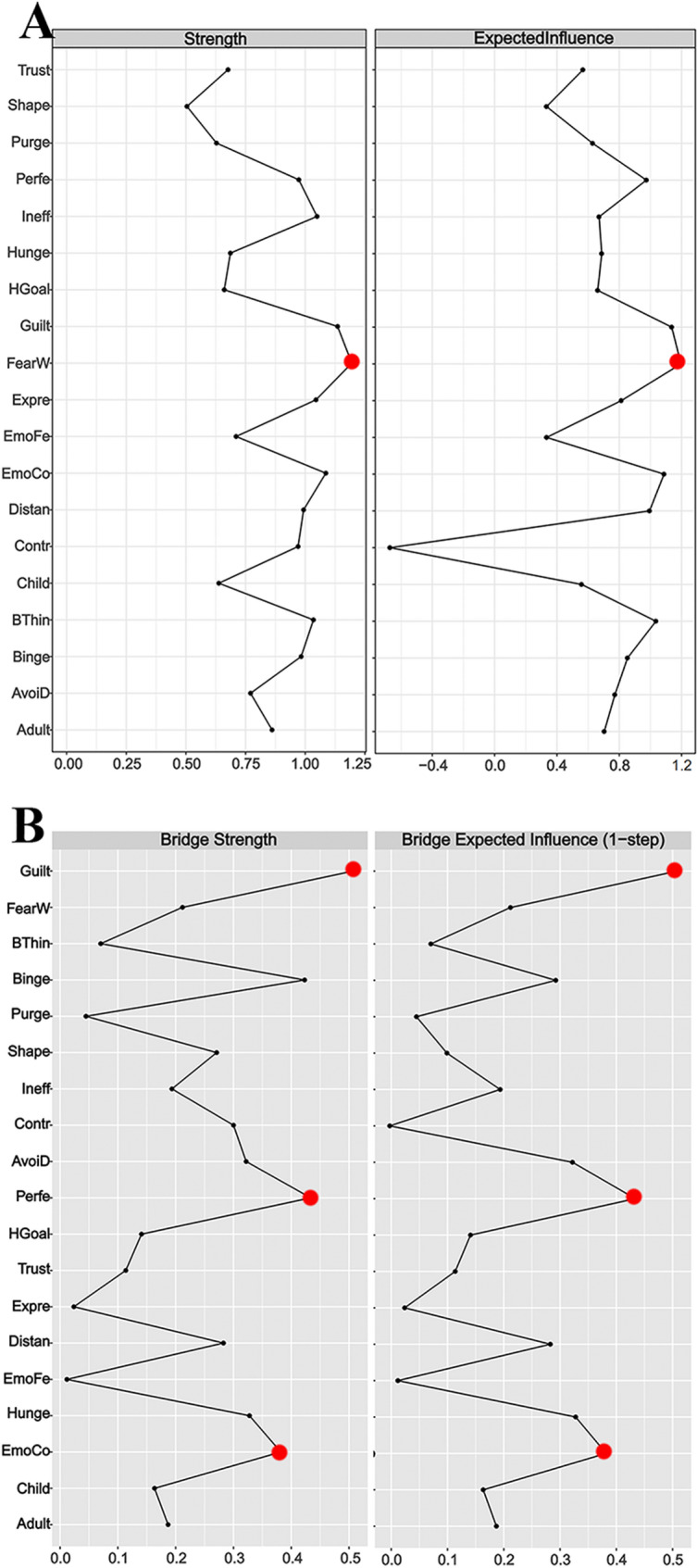
**A**: The strength and expected influence indices of the nodes in the network. **B**: The bridge strength and bridge expected influence indices of the nodes in the network

**Fig. 6:**
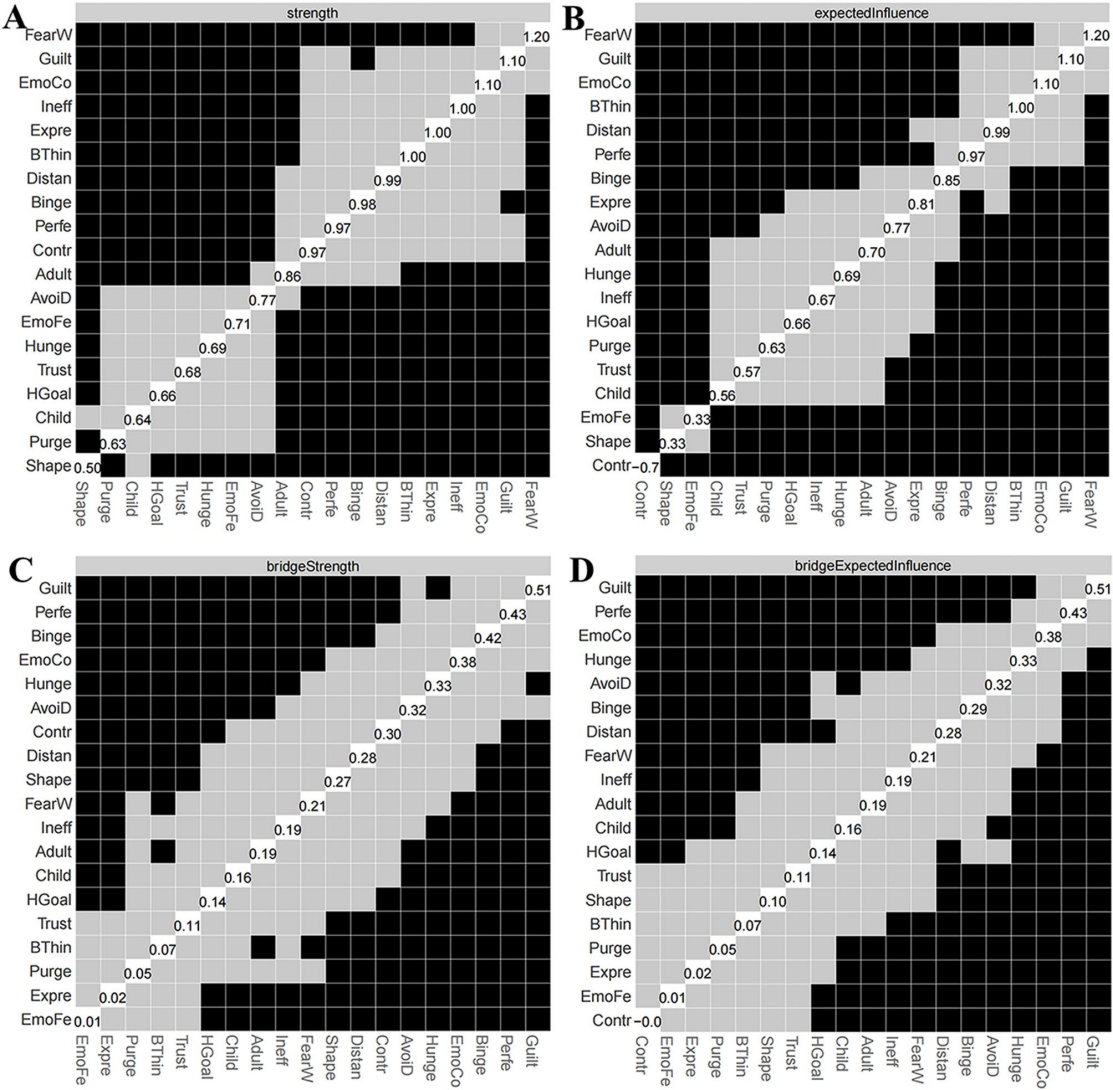
Bootstrapped difference test for (**A**) strength, (**B**) expected influence, (**C**) bridge strength, and (**D**) bridge expected influence in the network. The numbers in the white boxes (i.e., diagonal line) represent the values of (**A**) strength, (**B**) expected influence, (**C**) bridge strength, and (**D**) bridge expected influence. Gray boxes indicate the value that do not differ significantly from one another, while black boxes indicate the value that do differ significantly (*P*<0.05)

The node “FearW” was identified as the central node in the symptom network. This is because “FearW” not only had the highest strength and EI values (see Table 2**and** Fig. 5), but the strength and EI values of this node also showed statistically significant differences compared to more than half of the other nodes (*p* < 0.05, see Fig. 6).

**Table 2:** Item strength, Bridge strength, expected influence (EI), and Bridge EI values

No.	Dimension	Item abbreviation	Strength	EI	Bridge strength	Bridge EI
**1**	**ED-specific**	Guilt	1.14	1.14	**0.51**	**0.51**
**2**		FearW	**1.20**	**1.20**	0.21	0.21
**3**		BThin	1.03	1.03	0.07	0.07
**4**		Binge	0.98	0.85	0.42	0.29
**5**		Purge	0.63	0.63	0.04	0.04
**6**		Shape	0.50	0.33	0.27	0.10
**7**	**Non-specific**	Ineff	1.05	0.67	0.19	0.19
**8**		Contr	0.97	-0.67	0.30	>-0.01
**9**		Trust	0.68	0.57	0.11	0.11
**10**		Expre	1.04	0.81	0.02	0.02
**11**		Distan	0.99	0.99	0.28	0.28
**12**		EmoFe	0.71	0.33	0.01	0.01
**13**		Hunge	0.69	0.69	0.33	0.33
**14**		EmoCo	1.09	1.09	**0.38**	**0.38**
**15**		Child	0.64	0.56	0.16	0.16
**16**		Adult	0.86	0.70	0.19	0.19
**17**	**Perfectionism**	AvoiD	0.77	0.77	0.32	0.32
**18**		Perfe	0.97	0.97	**0.43**	**0.43**
**19**		HGoal	0.66	0.66	0.14	0.14

Note: Guilt = guilty after overeating; FearW = terrified of gaining weight; BThin = desire to be thinner; Binge = could not stop eating binges; Purge = trying to vomit; Shape = feel satisfied with the shape; Ineff = feel ineffective as a person; Contr = feel generally in control; AvoiD = avoid disappointing parents and teachers; Perfe = must do things perfectly; HGoal = have extremely high goals; Trust = trust others; Expre = have trouble expressing emotions; Distan = keep people at a certain distance; EmoFe = clearly identify what emotion feeling; Hunge = get confused as to whether or not hungry; EmoCo = worry that feelings will get out of control; Child = return to the security of childhood; Adult = demands of adulthood are too great

### Bridge nodes

A total of three bridge nodes were identified, namely “Guilt”, “EmoCo”, and “Perfe”. These three nodes not only had the highest bridge strength and bridge EI values within their respective communities (see Table 2**and** Fig. 5), but the differences in bridge strength and bridge EI values of these nodes compared to most other nodes were also statistically significant (*p* < 0.05, see Fig. 6).

## Discussion

This study is the first to combine network analysis and EGA to identify key psychological characteristics in the symptom network of EDs in China. Through EGA, we divided the items into three communities: “ED-specific,” “Non-specific,” and “Perfectionism.” Subsequent network analysis identified four key nodes, namely the central node “terrified of gaining weight” and the bridge nodes “guilty after overeating,” “worry that feelings will get out of control,” and “must do things perfectly.”

### The drive for thinness appears to be a core feature in Chinese individuals with EDs

Two key psychological characteristics in this population— “terrified of gaining weight” and “guilty after overeating”—are both derived from the Drive for Thinness subscale, reflecting similarities with Western ED populations.

“Terrified of gaining weight” is identified as the most pivotal central symptom within the symptom network. Patients fearing weight gain may engage in avoidance behaviors, such as extreme dietary restriction or excessive exercise, paradoxically intensifying their fear and perpetuating the ED [38]. Consistent with our findings, previous research has also identified the fear of weight gain as a core symptom within the psychopathological network of EDs, and one of the most central fears associated with these conditions [39, 40]. Furthermore, studies have demonstrated that the fear of weight gain is closely linked to the prognosis of patients with EDs, underscoring its critical role in the disease state [41].

“Guilty after overeating” serves as the most critical bridge symptom. The guilt associated with overeating is strongly correlated with “terrified of gaining weight” and may perpetuate restrictive eating behaviors [42]. Previous network analyses have identified “guilty after overeating” as a central node within the symptom network [43, 44]. Furthermore, this study indicates that “guilty after overeating” is linked to a broad spectrum of nonspecific psychological characteristics in EDs. Given the undirected nature of the network, it remains unclear whether “guilty after overeating” is influenced by other nonspecific psychological characteristics of EDs or significantly influences their development. However, regardless of its specific relationships, this underscores the critical role of “guilty after overeating” in the pathology of EDs.

### Perfectionism may play a unique role in Chinese individuals with EDs

Interestingly, the items related to “Perfectionism” were categorized into the third community. According to the design of the EDI-1, ED-related psychological characteristics are generally divided into two categories: ED-specific and non-specific [22]. However, “Perfectionism” was neither grouped into the “Non-specific” community nor the “ED-specific” community. This suggests that perfectionism may play a unique role in the onset, maintenance, and comorbidity of EDs, and incorporating targeted interventions may yield better treatment outcomes [45, 46].

Research has indicated that communities detected at one point in time may remain consistent over time, potentially revealing stable patterns in symptom structures and leading to hypotheses about the etiological mechanisms generating and/or maintaining psychopathology [47]. Perfectionism may have a relatively unique etiological mechanism in EDs, which may explain why it remains independent from traditional community classifications. Additionally, perfectionism is considered a transdiagnostic characteristic across mental disorders and is strongly associated with anxiety and obsessive-compulsive disorders [45]. Given the high comorbidity rates between EDs and anxiety or obsessive-compulsive disorders [48, 49], it is necessary to explore the role of perfectionism in comorbidity, which may offer insights into addressing the comorbidity challenge [50].

“Must do things perfectly” is the key bridge node connecting the ED-specific and non-specific communities. This item pertains to self-oriented perfectionism, which involves striving to meet unrealistically high personal standards [51]. Our results indicate that self-oriented perfectionism, rather than socially prescribed perfectionism (e.g., “avoid disappointing parents and teachers”), is associated with broader ED psychopathology, which is consistent with previous research findings [52]. Additionally, a longitudinal study has shown that perfectionism, particularly self-oriented perfectionism, can predict abnormal eating behaviors [53]. These findings suggest that self-oriented perfectionism may play a crucial role in the development and maintenance of EDs.

### Emotional regulation difficulties may also be important for Chinese ED patients

“Worry that feelings will get out of control” represents the most critical non-specific psychological characteristic connected with other communities. This symptom, part of the Interoceptive Awareness subscale, reflects patients’ concerns over their emotional regulation abilities, suggesting an underlying deficiency [22]. Emotional regulation involves recognizing and adjusting emotions [54]. Emotional dysregulation is frequently linked to more severe symptoms of EDs [55]. Research indicates that difficulties in emotional regulation constitute a key trans-diagnostic psychological characteristic, potentially linked to the persistence of ED symptoms [56, 57]. Targeted interventions have been shown to significantly improve this symptomatology [58]. Furthermore, previous network analyses have corroborated “worry that feelings will get out of control” as a central symptom in EDs [13].

Although studies have highlighted that interoceptive deficits may be a trans-diagnostic key characteristic of EDs, the assessment tools used in these studies do not accurately reflect these deficits [8, 16, 59, 60]. For example, the EDI-IA subscale, commonly used in research, assesses only interoceptive deficits related to emotion and hunger, with a single dimension. Additionally, the multi-dimensional assessment tools used in some studies, such as the multidimensional assessment of interoceptive awareness, are not specific to EDs, which may not precisely reflect the interoceptive deficits of EDs [16]. However, in recent years, scholars have developed and validated eating disorder-specific interoceptive perception questionnaire specifically for EDs [61], which may be suitable for exploring the relationship between interoceptive deficits and ED symptoms in these patients.

## Limitations & future research

This study has several important limitations. First, the use of the EDI-1, rather than the more recent EDI-2 or EDI-3, was due to the lack of validated Chinese versions of the latter at the time of study initiation. Although the EDI-1 captures most core constructs of eating disorders, this choice may have influenced the identification of culturally specific psychological features and limited comparability with studies using the more recent EDI-2 or EDI-3. Second, participants were recruited from a hospital-based treatment center, which may limit the generalizability of the findings to individuals with EDs who are undiagnosed, untreated, or not seeking clinical care. Third, although the exclusion of individuals with comorbid major depressive disorder was intended to ensure the reliability of self-reported data, this decision may have narrowed the clinical scope of the sample. Fourth, due to sample size constraints, subgroup analyses by age or diagnostic category were not conducted. It is possible that the centrality of specific symptoms—such as perfectionism or fear of weight gain—may differ between subgroups. In our case, dividing the sample would have resulted in insufficient statistical power to estimate stable networks, given the number of nodes and the complexity of the model. As such, the applicability of the results to specific clinical subgroups remains uncertain. Fifth, the study focused on item-level psychological characteristics within the EDI framework, which may have overlooked broader domains relevant to ED psychopathology. Finally, the cross-sectional nature of the data precludes causal inference and limits the ability to assess changes in symptom structure over time. These limitations should be considered when interpreting the findings and designing future studies.

Future treatment strategies may need to be adjusted based on the key characteristics of EDs. Our study suggests that, in addition to targeting drive for thinness, interventions for the psychotherapy of EDs should also focus on interoceptive deficits and perfectionism as key areas for treatment. For instance, CBT-E helps patients address distorted perceptions of body shape and weight and reduce the morbid drive for thinness [62]. Incorporating modules such as perfectionism and emotional intolerance may further enhance therapy effectiveness in treating Chinese ED patients [46, 63]. In addition, the incorporation of above targeted modules as adjunctive components within family-based treatment has the potential to enhance remission rates among underage patients with EDs [64]. Other therapeutic approaches that can directly address perfectionism and emotional intolerance, such as compassion-focused therapy, may also be integrated with standard treatment frameworks to improve overall clinical outcomes [65].

## Conclusion

To our knowledge, this study is the first to combine network analysis and EGA to investigate key psychological characteristics of EDs in the Chinese population. The findings suggest that, in addition to drive for thinness, perfectionism and emotional regulation difficulties may represent key characteristics in this population. Further research is warranted to confirm these findings and support the development of more targeted and effective treatment strategies.

## Data Availability

No datasets were generated or analysed during the current study.

## Abbreviations

EDs: Eating disorders
ED-RP: Eating disorder registration project
EDI-1: Eating disorder inventory-1
CBT-E: Enhanced cognitive behavior therapy
SD: Standard deviation
EGA: Exploratory graph analysis
EI: Expected influence
CS: Correlation stability
